# The Nutraceutical Properties of “Pizza Napoletana Marinara TSG” a Traditional Food Rich in Bioaccessible Antioxidants

**DOI:** 10.3390/antiox10030495

**Published:** 2021-03-22

**Authors:** Irene Dini, Luana Izzo, Giulia Graziani, Alberto Ritieni

**Affiliations:** 1 Department of Pharmacy, University of Naples Federico II, Via Domenico Montesano 49, 80141 Napoli, Italy; luana.izzo@unina.it (L.I.); giulia.graziani@unina.it (G.G.); alberto.ritieni@unina.it (A.R.); 2 UNESCO Chair of Health Education and Sustainable Development, University of Naples, 80131 Napoli, Italy

**Keywords:** Pizza Napoletana, lycopene, polyphenols, bioaccessibility, nutraceutical properties, antioxidant activity

## Abstract

Italian gastronomy experiences have ever-enhancing fame around the world. It is due to the linkage between taste and salubriousness commonly related to Mediterranean foods. The market proposes many types of pizza to suit all palates. The antioxidant potential of the “Pizza Napoletana marinara” included in the register of traditional specialties guaranteed (TSG) was determined in this work. ABTS (2,2’-azino-bis(3-ethylbenzothiazoline-6-sulfonic acid) method evaluated the antioxidant activity of the pizza homogenized. In vitro digestion models estimated the intestinal and gastric bioaccessibility of the main antioxidant compounds (lycopene and phenolics). To our knowledge, this is the first study to provide the content, antioxidant potential, and bioaccessibility of the antioxidants (polyphenols and lycopene) contained in the traditional pizza “marinara TSG”. Our results showed that the “Pizza Napoletana marinara” had polyphenols concentration, lycopene level, antioxidant activity, and bioaccessibility of phenolic compounds and lycopene better than other similar pizzas. They confirmed the nutritional importance of traditional preparations and established the nutraceutical potential of “pizza marinara TSG” as a food rich in bio-accessible antioxidants.

## 1. Introduction

Italian gastronomy experiences have ever-enhancing fame around the world. It is due to the linkage between taste and salubriousness commonly related to Mediterranean foods. Pizza is a universal, appreciated product of Italian gastronomy. It was cooked in southern Italy for the first time (before 1000 AD). Pizza was created for poor people with inexpensive ingredients flour, yeast, water, edible oil, and salt. In the 16th century, when the tomato was imported from America, pizza became as we know it today [1]. In 1750, the first “marinara” (tomato, garlic, oregano, oil) was made, and in 1850 the first “Margherita” (tomato, mozzarella, oil) was cooked [1]. In 1889, the pizza maker Raffaele Esposito added the basil to the pizza “Margherita”, giving it the Italian flag colors [1]. Recently, the food market has grown, offering novel chances to catering. Pizza has become an international food. Several imitations have been made. New kinds of pizzas demand their own identities (e.g., crispy covered with tomato sauce, shredded cabbage, caramelized red onions, sundried tomatoes, spinach, etc.). They often have nothing to do with Neapolitan pizza, which is a result of traditional Mediterranean culinary knowledge. These pizzas differ from the original product in both organoleptic and nutritional characteristics. Some products are made with cheap flour, oil, and tomatoes and damage the traditional product’s reputation. Traditional foods represent the identity, culture, history, local economy, and heritage and are essential elements for a country’s dietary patterns. Pizza has a crucial role in the traditional habits of Italian culture. Several certificates and labels have been assigned to preserve the Neapolitan pizza identity. The name “Pizza Napoletana” has been entered in the register of Traditional Specialties Guaranteed (TSG) [2]. TSG is a tolerant European Union (EU) food designation. It designates foods made with “traditional” techniques or ingredients with proven usage in the Community market for at least 25 years. TSG makes the product distinguishable from other similar products, and the consumers are informed based on its characteristics. In 2015, the Italian Ministry of Agricultural, Food, and Forestry Policies added Neapolitan pizza to the list of traditional agri-food products [3]. The “Pizza Napoletana” is an elastic, soft, and easily foldable food with a distinctive savory. Due to the typical taste of well-cooked bread, tomato, baked mozzarella, and flavor (garlic, oregano, basil). The name “Pizza Napoletana” is limited to “Pizza Napoletana marinara” (tomato, oregano, extra virgin olive oil, and garlic) and “Pizza Napoletana Margherita” (tomato, grated cheese, mozzarella or fior di latte, extra virgin olive oil, and basil) pizzas made according to precise guidelines and with traditional ingredients. Flour, yeast, water, edible oil, and salt are kneaded. The dough ferments twice. The first fermentation lasts 2 h; then, the dough is portioned into balls that ferment for another 4–6 h at room temperature. Successively the balls are stretched with the hands, guaranteeing to leave a denser edge on the outer part. Finally, the toppings are added, and the pizza is baked in a wood oven at 485 °C for 60–90 s. The cooking methods of these foods have been handed down from generation. Lifestyle changes are affecting eating habits, and new pizza is changing from the traditional product. Some traditional preparations have shown health properties that have been tested over time [4,5]. An indicator of food preparation’s potential benefits is the antioxidant activity of food compounds and their possible synergistic interactions. The antioxidant potential of food is linked to phenolic compounds, carotenoids, and vitamins (C and E). Natural antioxidants have anti-inflammatory, antioxidant, anti-allergic, anti-atherogenic, antithrombotic, cardioprotective, and antimicrobial effects [6,7]. Nutraceutical values are useful parameters to promote culinary products. Nutraceutical data of composite foods are essential to prepare the basis of dietary recommendations. There are no data about the nutraceutical potential of pizza. The antioxidant data of composite foods are often calculated from the corresponding individual ingredients and do not consider the transformations produced by cooking techniques, or, above all, what happens in the human organism and the real bioaccessibility of these molecules. It is essential to investigate these compounds’ possible interactions and the bioaccessibility within our body to get an idea of their possible beneficial effects. The antioxidant potential of both “Pizza Napoletana” principally depends on the bioavailability of the phenolic compounds and lycopene in extra virgin olive oil (EVOO) and tomatoes. The pizzerias that do not follow the “Pizza Napoletana” production specification replace EVOO with other less expensive oils such as other vegetable oils or olive oil, a mixture of refined olive oil and virgin olive oil. The type of oil used influences not only the sensorial [8,9] and technological properties [10], but also affects the nutritional and nutraceutical properties of the pizza [11]. The oil composition differs in the profile and content of unsaturated fatty acids and antioxidant molecules. During cooking, unsaturated fatty acids oxidase differently. Compared to other oil, EVOO shows higher resistance to lipid oxidation for the presence of polyphenols [12,13,14,15,16]. Thermal oxidation of heated oils produces free radicals associated with the pathogenesis of many diseases, including cardiovascular diseases and atherosclerosis [17,18,19,20,21,22,23,24]. Tomato is another essential ingredient of pizza. Tomato sauce is used to prepare traditional pizza TSG. New pizzas are made both with fresh cherry tomatoes and tomato sauce. The treatment of the tomatoes to produce tomato sauce modifies the physicochemical attributes (color, viscosity, total soluble solids) [22], the pH, and some product quality parameters such as lycopene content in the tomato sauce [25,26,27]. Lycopene is a carotenoid with antioxidant properties that can decrease the risk of hypercholesterolemia, atherosclerosis, cancer, osteoporosis, infertility, metabolic syndrome, and liver damage [28,29]. In this work, the lycopene level, the antioxidant activity, and the bioaccessibility of lycopene and polyphenols in “Pizza Napoletana marinara” and other similar pizzas not subjected to the production disciplinary were detected to highlight the nutraceutical properties of the pizza “marinara TSG”.

## 2. Materials and Methods

The pizza “marinara TSG” was prepared according to the production disciplinary of the authentic Neapolitan Pizza Association called “Associazione Verace Pizza Napoletana” or A.V.P.N [30]. 

The other pizzas like pizza “marinara TSG” varied for oil type (soybean oil, sunflower oil, and olive oil) and tomato sauce or fresh tomato (cherry tomato) used in the recipe. The pizzas were cooked in a wood oven, weighed, homogenized (Ultra Turrax T25 homogenizer, IKA-Werke, Wilmington, NC, USA), and stored at −18 °C until the analysis.

### 2.1. Chemicals

Chemicals and enzymes were bought from Sigma Aldrich (St. Louis, MO, USA) unless specified differently. Artificial saliva was obtained by mixing KCl (89.6 g/L), KSCN (20 g/L), NaH_2_PO_4_ (88.8 g/L), NaSO_4_ (57 g/L), NaCl (175.3 g/L), NaHCO_3_ (84.7 g/L), urea (25 g/L), and α-amylase (290 mg) in 80 mL purified water. The pH of the solution was adjusted to 2 with HCl 6 N [30]. 

### 2.2. Total Phenolics Extraction and Quantification

We extracted 3 g of homogenized pizza with 30 mL of methanol/water (70:30, *v/v*). The extraction procedure was repeated twice for each sample. The mixtures were centrifuged at 4000× *g* rpm in a centrifuge professional mod. N.E.Y.A. 10 (Neya Centrifuges, Carpi, Italy), filtered through a Whatman filter paper (Whatman^®^ filters, Global Life Sciences Solutions, Marlborough, MA, USA) and then used for antioxidant activity assay. Total polyphenol content was measured using the Folin–Ciocalteu colorimetric method [31]. Polyphenolic extracts (0.1 mL) were mixed with Folin–Ciocalteu reagent (0.2 mL) and H_2_O (2 mL) and incubated at room temperature for 3 min. Successively, 20% sodium carbonate (1 mL) was added to the mixture, and after 1 h of incubation at room temperature, the total polyphenols were determined spectrophotometrically (λ 765 nm) in a spectrophotometer (Lambda 25, PerkinElmer, Italy). The results were expressed as gallic acid equivalents (G.A.E.), milligrams per 100 g of sample. All determinations were performed in triplicate (*n* = 3) (Figure S2; Tables S1 and S2).

### 2.3. Antioxidant Activity

The antioxidant assay was performed by the ABTS method [32]. ABTS (7 mM) and potassium persulfate (2.45 mM) were left at room temperature (23 °C) in the dark for 16 h. The filtered sample was diluted with 70% methanol giving 20–80% inhibition of the blank absorbance with a 0.1 mL sample. ABTS solution (1 mL; absorbance = 0.700 ± 0.050) was added to 0.1 mL of the tested samples and mixed thoroughly. The reaction mixture was left at room temperature for 2.5 min, and successively the absorbance was measured at λ 734 nm in a spectrophotometer (Lambda 25, PerkinElmer, Italy). Trolox standard solution (final concentration 0–15 M) in methanol was assayed at the same conditions. The results were expressed as Trolox equivalent antioxidant capacity (T.E.A.C., mmol Trolox equivalents) on 100 g of sample.

### 2.4. Lycopene Extraction and Quantification

The homogenized sample (6 g) was extracted with 100 mL of hexane in an orbital shaker (Infors AG CH-4103, Bottmingen, Switzerland) for 2 min at 21,500 rpm. Extraction was repeated until the residue was devoid of color. The extracts were centrifuged at 4000× *g* rpm for 5 min. in a centrifuge professional mod. N.E.Y.A. 10 (Neya Centrifuges, Carpi, Italy). The supernatants were filtered with RC 0.45 μm microfilters (Whatman^®^ regenerated cellulose membrane filters, Global Life Sciences Solutions, Marlborough, MA, USA). Lycopene was quantified spectrophotometrically (λ 502 and λ 472 nm) in a spectrophotometer (Lambda 25, PerkinElmer, Italy) (Figure S1) [29].

### 2.5. Digestion Procedure

All samples were subjected to the in vitro digestion model [33]. Each sample was mixed with saliva/pepsin/HCl digestion for 2 h at 37 °C.

### 2.6. Duodenal Digestion Simulation

The samples were mixed with 6 mL of artificial saliva (immediately after its preparation), 0.5 g of pepsin (14,800 U) and HCl 0.1 N, incubated for 2 h at 37 °C, and blended in an orbital shaker (Infors AG CH-4103, Bottmingen, Switzerland) at 55 rpm [34].

### 2.7. Pancreatic Digestion Simulation

The sample pH was brought to 6.5 with NaHCO_3_ 1 N and successively added with 5 mL (1:1; *v/v*) of pancreatin (8 mg/mL), bile salts (50 mg/mL), and water (20 mL). The solution was incubated at 37 °C for 2 h and blended in an orbital shaker (Infors AG CH-4103, Bottmingen, Switzerland) at 55 rpm. Of the mixture, 30 mL was centrifuged at 4000× *g* rpm at 4 °C for 1 h in a centrifuge professional mod. N.E.Y.A. 10, (Neya Centrifuges, Carpi (MO), Italy). The supernatant (bio-accessible fraction) was collected, and the concentration of the lycopene and the total phenolics were evaluated according to the methods described previously [34].

### 2.8. Statistical Analysis

Significant differences between mean values were determined by performing a one-way ANOVA test (significant level *p* < 0.05). Correlation coefficients were calculated using Pearson product-moment correlation (*ρ*). Excel spreadsheet software, version 19.0 (Microsoft, Redmond, WA, USA) was used to perform statistical analyses.

## 3. Results

Levels, antioxidant activity, and bioaccessibility of lycopene and polyphenols were evaluated in pizzas prepared with oils and tomatoes of different qualities.

### 3.1. Total Phenolics Quantification

**Figure 1:**
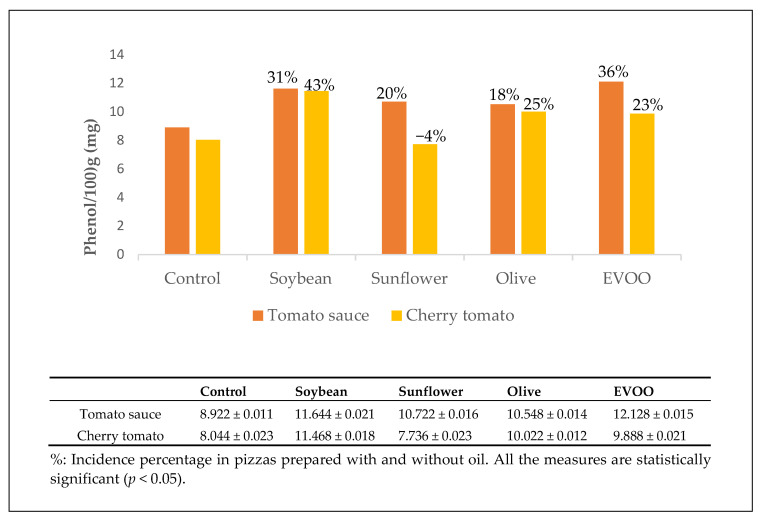
Amount of phenols expressed as mg/100 g of pizza measured on lipophilic extracts as a function of the type of oil and tomato used.

The first step was the evaluation of the concentration of phenols in pizza “marinara” made with similar procedures (fermentation of dough and cooking modality), but with different oil (soybean, sunflower, olive, and EVOO) and tomato quality (sauce and cherry tomatoes). Then the results were compared with those of pizzas prepared without oil (control). The addition of oil increased the content of phenolic compounds in all the “marinara” pizzas, except for the pizza prepared with sunflower and cherry oil (Figure 1). The “vera Pizza Napoletana marinara” TSG showed a content of phenolic compounds higher than that of the other pizzas.

### 3.2. Antioxidant Activity

**Figure 2:**
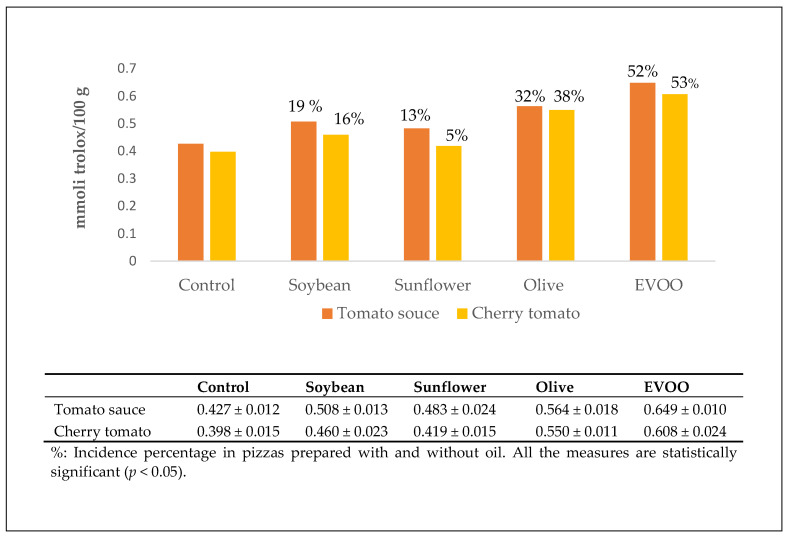
Antioxidant activity of the polyphenolic extracts.

The addition of oil increased the antioxidant activity of the pizzas. The “vera Pizza Napoletana marinara” TSG had the highest potential (Figure 2).

### 3.3. Correlation between Antioxidant Activity Assays and Total Phenolic Content

The Pearson’s product-moment correlation coefficients (*ρ*) between antioxidant activity assays and total phenolic content were calculated. Significant positive correlations were observed between pizzas made with tomato sauce antioxidant activity and total phenolic content (*ρ* = 0.79; *p* < 0.05) and between pizzas made with cherry tomato antioxidant activity and total phenolic content (*ρ* = 0.50; *p* < 0.05). 

### 3.4. Bioaccessibility of the Phenolic Compounds

Finally, the phenolic compounds’ bioaccessibility at gastric and intestinal levels was tested (Table 1). Gastric digestion increases the bioaccessibility of total polyphenols in pizzas made with oil. The transition from the gastric (acid environment) to the intestinal (mild alkaline environment) caused a much greater increase in the bioaccessibility of total polyphenols, especially those contained in “vera Pizza Napoletana marinara” TSG.

### 3.5. Correlations between Polyphenols in the Pizzas and Polyphenols Released in the Gastric and Intestinal Phases

The Pearson’s product-moment correlation coefficients between polyphenols content in the pizzas and polyphenols released in the gastric and intestinal phases were calculated. Significant positive correlations were observed between polyphenol content in the pizzas and polyphenols released in the gastric (*ρ* = 0.35; *p* < 0.05) and intestinal phases (*ρ* = 0.70; *p* < 0.05). A significant negative correlation was observed between polyphenol content in the pizzas and polyphenols in the pellet (*ρ* = −0.65; *p* < 0.05).

### 3.6. Correlations between Polyphenols in the Pizzas Made with Tomato Sauce and Polyphenols Released in the Gastric and Intestinal Phases

The Pearson’s product-moment correlation coefficients between polyphenol content in the pizzas made with tomato sauce and polyphenols released in the gastric and intestinal phases were calculated. Significant positive correlations were observed between polyphenols in the pizzas and polyphenols released in the gastric (*ρ* = 0.87) and intestinal phases (*ρ* = 0.88; *p* < 0.05). A significant negative correlation was observed between polyphenols in the pizzas and polyphenols in the pellet (*ρ* = −0.93; *p* < 0.05).

### 3.7. Correlations between Polyphenols in the Pizzas Made with Cherry Tomato and Polyphenols Released in the Gastric and Intestinal Phases

The Pearson’s product-moment correlation coefficients between polyphenols content in the pizzas made with cherry tomato and polyphenols released in the gastric and intestinal phases were calculated. Significant positive correlations were observed between polyphenols in the pizzas made with cherry tomato and polyphenols released in the gastric (*ρ* = 0.61; *p* < 0.05) and intestinal phases (*ρ* = 0.56; *p* < 0.05). A significant negative correlation was observed between polyphenols in the pizzas made with cherry tomato and polyphenols in the pellet (*ρ* = −0.58).

### 3.8. Lycopene Content

**Figure 3:**
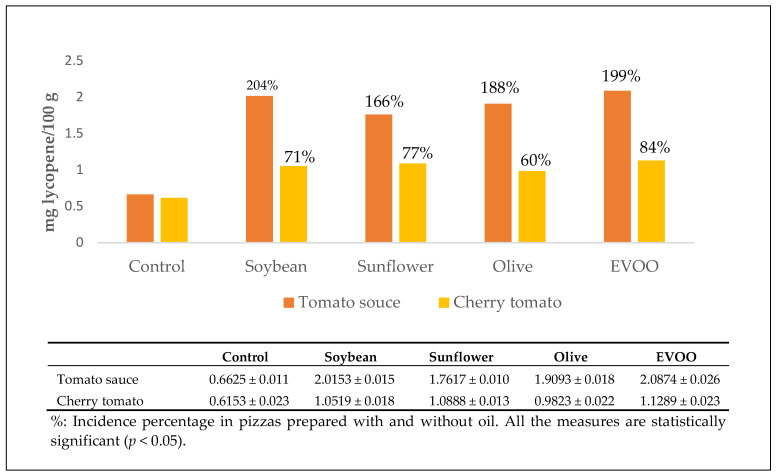
Amount of lycopene expressed as mg/100 g of pizza measured on lipophilic extracts as a function of the type of oil used.

The pizzas prepared with oil contain high lycopene levels. The “vera Pizza Napoletana marinara” TSG showed the highest lycopene content (Figure 3).

### 3.9. Antioxidant Activity of the Carotenoid Extracts

**Figure 4:**
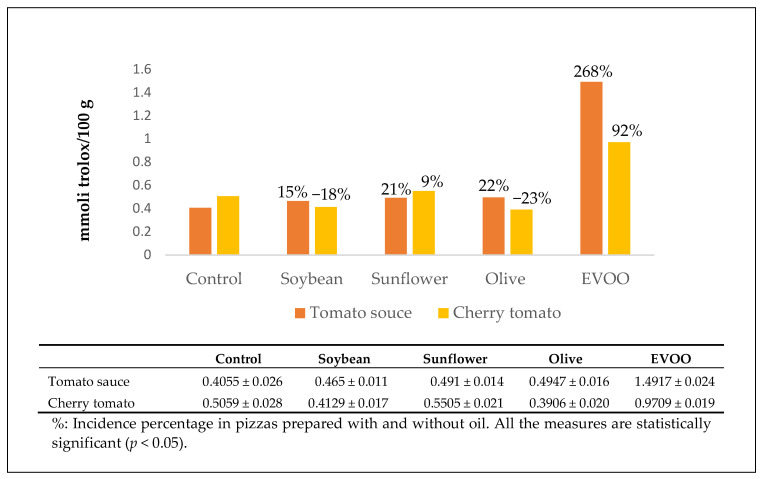
Antioxidant activity of the carotenoid extracts.

The “vera Pizza Napoletana marinara” TSG had the highest antioxidant potential (Figure 4).

### 3.10. Correlations between Antioxidant Activity Assays and Total Lycopene Content

The Pearson’s product-moment correlation coefficients between antioxidant activity assays and total phenolic content were calculated. Significant positive correlations were observed between pizzas made with tomato sauce antioxidant activity and total lycopene (*ρ* = 0.44; *p* < 0.05) and the pizzas made with cherry tomato antioxidant activity and total lycopene (*ρ* = 0.40; *p* < 0.05). 

### 3.11. Bioaccessibility of the Lycopene

The bioaccessibility of lycopene was reported in Table 2. Lycopene was released both gastrically and intestinally. As already happened for polyphenols, the acid pH favored the release more. Unlike polyphenols, however, a high concentration of lycopene remained undigested. The lycopene contained in the “marinara TSG” pizzas was the most bioaccessible.

### 3.12. Correlations between Lycopene in the Pizzas and Lycopene Released in the Gastric and Intestinal Phases

The Pearson’s product-moment correlation coefficients between lycopene in the pizzas and lycopene released in the gastric and intestinal phases were calculated. Significant positive correlations were observed between lycopene in the pizzas and lycopene released in the gastric (*ρ* = 0.21) and intestinal phases (*ρ* = 0.72). A significant negative correlation was observed between lycopene content in the pizzas and lycopene in the pellet (*ρ* = −0.51).

### 3.13. Correlations between Lycopene in the Pizzas Made with Tomato Sauce and Lycopene Released in the Gastric and Intestinal Phases

The Pearson’s product-moment correlation coefficients between lycopene in the pizzas made with tomato sauce and lycopene released in the gastric and intestinal phases were calculated. Significant positive correlations were observed between lycopene content in the pizzas made with tomato sauce and lycopene released in the gastric (*ρ* = 0.83; *p* < 0.05) and intestinal phases (*ρ* = 0.93; *p* < 0.05). A significant negative correlation was observed between lycopene content in the pizzas made with tomato sauce and lycopene in the pellet (*ρ* = −0.90; *p* < 0.05).

### 3.14. Correlations between Lycopene in the Pizzas Made with Cherry Tomato and Lycopene Released in the Gastric and Intestinal Phases

The Pearson’s product-moment correlation coefficients between lycopene in the pizzas and lycopene released in the gastric and intestinal phases were calculated. Significant positive correlations were observed between lycopene in the pizzas made with cherry tomato and lycopene released in the gastric (*ρ* = 0.63; *p* < 0.05) and intestinal phases (*ρ* = 0.81; *p* < 0.05). A significant negative correlation was observed between lycopene in the pizzas made with cherry tomato and lycopene in the pellet (*ρ* = −0.73; *p* < 0.05).

## 4. Discussion

In recent years, there has been a new interest in traditional cuisine and traditional foods [35] rich in antioxidant compounds, which are useful for preventing some chronic-degenerative diseases that cause death in our society, such as cardiovascular diseases and cancer [36]. Crucial nutritional information to define the population’s daily intake and their association with health effects is determined by antioxidant compound content and their bioavailability [37]. This study compares the concentrations of some parameters of nutraceutical interest present in pizza “marinara TSG” and other pizzas called pizza “marinara” but not prepared according to the production disciplinary of TSG. Making Neapolitan pizza TSG is an art that respects the use of indicated ingredients, techniques, and methodologies strictly regulated. The preparation of the pizza varies according to the typology of oil and tomato used. These two products influence the pizzas’ nutrient profile as they bring different concentrations of polyunsaturated fatty acids, phenolics, carotenoids, and other compounds useful for human health. The “Pizza Napoletana” is made exclusively in wood ovens at 485 °C for 60–90 s [2]. The temperature reached by pizza is ~204–288 °C [2]. The thermal process produces lipid oxidation, caramelization, and Maillard reaction. The unsaturated fatty acids contained in the vegetable oils oxidize and change their compositions and nutritional value. The oxidation of fatty acids depends on the fatty acid composition, polyphenols profile, and content [38]. In this work, the addition of oil in the pizza positively contributed to the total polyphenol content. The polyphenolic content and antioxidant activity of “Pizza Napoletana marinara” TSG was superior to other pizzas. The phenolic composition of the oils used to make pizzas was very variable. In the EVOO, there are five different classes of phenolic compounds (secoiridoid, phenolic alcohols, phenolic acids, and flavonoids) [37]. Of these, the secoiridoid were the most abundant [39]. During the cooking, the secoiridoids hydrolyzed. Nevertheless, pizza’s antioxidant potential remains high as their hydrolysis products (tyrosol and hydroxytyrosol) have antioxidant activity [40]. In vitro tests determined the phenolic concentrations that are absorbed and available for physiological functions. Bioaccessibility is an essential prerequisite for bioavailability. It indicates the level of bioactive compounds solubilized in chyme (supernatant) after each step of digestion and potentially available for absorption. In vitro digestion simulates human gastric and intestinal digestion. In vitro tests are faster, less expensive, and offer better controls of experimental variables than in vivo studies [41]. The gastric digestion simulation was obtained, adding pepsin and acidifying the samples to pH 2 (an adult’s gastric pH). The acidification of the samples prevents the denaturation of pepsin that occurs at pH ≥ 5 [42]. The intestinal digestion was mimicked by neutralizing the sample (pH 5.5–6), adding pancreatin and bile salts (emulsifiers), and finally readjusted to pH 6.5. The polyphenols in the pizzas were released only in small quantities at the gastric level, as shown in correlation studies. Pizzas, like all solid matrices, release phenolic compounds with difficulty. It is necessary to extract them to increase their bioaccessibility and potentially bioavailability. The intestine’s alkaline pH improves the extraction of phenolic compounds more than the gastric’s acidic pH. The bioaccessibility of the phenolic fraction of pizza “marinara TSG” was higher than other similar pizzas [43]. The potential nutraceutical properties of pizza are linked to the tomato quality and the lycopene it contains [44,45]. Correlation studies showed that the lycopene’s bioaccessibility improves when tomatoes are turned into sauce since heat treatment destroys the cell wall membrane improving lycopene release [46] and denaturing carotene–protein interactions. [47,48]. In pizzas, the rapid heat treatment does not allow the complete breakage of the membrane, but the formation of micelles with the oil lipids facilitates the lycopene release. Therefore, the type of tomato is not an ancillary ingredient. The better choice is the tomato sauce instead of cherry tomato. The sauce may be considering predigested and the final sauce is more bioavailable by the fat of vegetal oil than the untreated cherry tomato. In this work, the carotenoid extract’s antioxidant activity increased when pizza was prepared with oil addition, and it was higher in the pizza “Pizza Napoletana marinara” TSG. Improvement of antioxidant activities in processed foods could be linked to the botanicals released from the matrix during processing [49], the formation of the Maillard products [50], deactivation of the endogenous oxidative enzymes, and polymerization due to heating [51]. This result was probably due to the mutual protection of tomato and olive oil in the “Pizza Napoletana marinara”, rich in antioxidant compounds [52]. The oil on the pizzas products dissolves the lycopene in tomato, insoluble in the crystalline form [53]. Moreover, during the cooking process, the flavonoid glycosides of the EVOO, form free hydroxyl phenol [54,55,56] to protect lycopene from thermal oxidation. Lycopene’s bioaccessibility level was higher in “Pizza Napoletana marinara” TSG than other similar products. The bile acids and the pancreatin contribute to the lycopene’s absorption, incorporating it into micelles and making it available for absorption [57]. The quality of the oil used to prepare pizza had an impact on the lycopene bioaccessibility. The EVOO resulted in the highest lycopene bioaccessibility. Lipids rich in C12:0 fatty acids determine the lycopene’s lower bioaccessibility than lipids containing many 18:1 fatty acids (EVOO and olive oil). The first fatty acids form with monoglycerides, weakly swollen micelles, making lycopene not very soluble [58]. Moreover, the EVOO offers the necessary environment for the isomerization of the lycopene [59]. Short cooking time and the use of the EVOO enhance the lycopene Z-isomers formation [60]. The *Z*-isomers have absorption, transport flexibility, and antioxidant capacity higher than *E*-isomers [61]. This hypothesis follows a previous study, which show that the co-digestion of tomato sauce with different added oils caused a higher lycopene bioaccessibility when EVOO was added [62]. Moreover, Tulipani et al. [63] hypothesized that the lipid matrix in the sauces might stimulate the re-absorption events by enterohepatic circulation, potentially affecting the apparent plasma half-life of these compounds. Significant positive correlations between antioxidant activity, phenolic content, and lycopene suggest that phenols and lycopene in pizzas prepared using tomato sauce and cherry tomato can chelate ferrous ions. Both correlation values being higher in pizzas prepared with tomato sauce suggested that the tomato’s transformation into sauce positively impacted this activity. The negative correlation between the polyphenols in the pizzas and the pellets when using vegetable oils, especially adopting EVOO, confirms that the main part of the health compounds is captured and absorbed by the intestinal wall and put in the blood flow. Importantly, polyphenols’ adsorption rate is better when pizza is baked with EVOO compared to other vegetable oils or control samples.

## 5. Conclusions

This study investigated the nutraceutical potential of the “Pizza Napoletana marinara” TSG. For the first time, the concentration of polyphenols and lycopene (well-known antioxidant molecules) in the “Pizza Napoletana marinara” TSG and their bioavailability in the human body was assessed. The results were compared with those obtained by analyzing the same parameters in pizza “marinara” prepared not following the TSG specification and without oil added. Our results show that the “Pizza Napoletana marinara” TSG had the highest antioxidant activity, polyphenols, and lycopene content than other “marinara” pizzas. Moreover, the mix of ingredients used for its preparation contributed to making the lycopene more bioavailable for our health. It appears very interesting that two unrelated pizza cooking steps, like tomato production and EVOO, synergically provide a more healthy and complete meal for consumers. It is not unexpected that the lycopene behavior is too similar to polyphenols since this antioxidant, considered a tomato health biomarker, has excellent solubility in fat or vegetable oils. Historic craft, the know-how of pizza baking, rigorous rules, and best-quality of local raw products are reliable drivers of the success of the traditional pizza “marinara TSG.”

Our results confirm the nutritional importance of traditional preparations and demonstrate the nutraceutical potential of the “Pizza Napoletana marinara” TSG as a food rich in bioavailable antioxidants. This data could be used to write a nutraceutical label on traditional pizza to help consumers make informed pizza selections.

## Figures and Tables

**Table 1:** Bioaccessibility of polyphenols in the “marinara” pizzas expressed as percentage released at the gastric and intestinal level.

Sample	Polyphenols/5 gr (mg) of Pizza	% Polyphenols Released (Gastric Phase)	% Polyphenols Released (Intestinal Phase)	% Polyphenols Residual Pellet
ControlT	0.4461 ± 0.021	5	46	48
ControlC	0.4022 ± 0.019	5	41	54
SoybeanT	0.5823 ± 0.034	10	69	21
SoybeanC	0.5734 ± 0.021	15	60	25
SunflowerT	0.5361 ± 0.012	12	60	28
SunflowerC	0.3868 ± 0.018	13	58	29
OliveT	0.5274 ± 0.017	9	71	20
OliveC	0.5010 ± 0.012	15	69	16
EVOOT	0.6064 ± 0.078	13	73	14
EVOOC	0.4944 ± 0.075	18	68	14

All the measures are statistically significant (*p* < 0.05). ControlT: pizza made with tomato sauce, oregano, and garlic; ControlC: pizza made with cherry tomato, oregano, and garlic; SoybeanP: pizza made with tomato sauce, oregano, garlic, and soybean oil: SoybeanC: pizza made with cherry tomato, oregano, garlic, and soybean oil; SunflowerT: pizza made with tomato sauce, oregano, garlic, and sunflower oil; SunflowerC: pizza made with cherry tomato, oregano, garlic, and sunflower oil; OliveT: pizza made with tomato sauce, oregano, garlic, and olive oil; OliveC: pizza made with cherry tomato, oregano, garlic, and olive oil; EVOOT (“pizza marinara” TSG): pizza made with tomato sauce, oregano, garlic, and EVOO; EVOOC: pizza made with cherry tomato, oregano, garlic, and EVOO.

**Table 2:** Bioaccessibility of lycopene in the “marinara” pizzas expressed as percentage released at the gastric and intestinal level.

Sample	Lycopene/5 gr (mg) of Pizza	% Lycopene Released (Gastric Phase)	% Lycopene Released (Intestinal Phase)	% LYCOPENE Residual Pellet
ControlT	0.03312 ± 0.002	9	16	75
ControlC	0.03076 ± 0.001	11	12	77
SoybeanT	0.08482 ± 0.003	15	26	59
SoybeanC	0.05259 ± 0.002	15	23	62
SunflowerT	0.08808 ± 0.001	13	26	61
SunflowerC	0.05443 ± 0.002	20	23	57
OliveT	0.09546 ± 0.004	22	33	45
OliveC	0.04911 ± 0.003	29	31	40
EVOOT	0.09907 ± 0.003	21	35	44
EVOOC	0.05644 ± 0.005	28	33	39

All the measures are statistically significant (*p* < 0.05). ControlT: pizza made with tomato sauce, oregano, and garlic; ControlC: pizza made with cherry tomato, oregano, and garlic; SoybeanP: pizza made with tomato sauce, oregano, garlic, and soybean oil: SoybeanC: pizza made with cherry tomato, oregano, garlic, and soybean oil; SunflowerT: pizza made with tomato sauce, oregano, garlic, and sunflower oil; SunflowerC: pizza made with cherry tomato, oregano, garlic, and sunflower oil; OliveT: pizza made with tomato sauce, oregano, garlic, and olive oil; OliveC: pizza made with cherry tomato, oregano, garlic, and olive oil; EVOOT (“pizza marinara” TSG): pizza made with tomato sauce, oregano, garlic, and EVOO; EVOOC. pizza made with cherry tomato, oregano, garlic, and EVOO.

## Data Availability

The data presented in this study are available in article and supplementary material.

## Supplementary Materials

The following are available online at https://www.mdpi.com/2076-3921/10/3/495/s1, Figure S1: Comparison from carotenoids profile of tomato sauce and cherry tomato: peak 1 (lutein), peak 2 (lycopene), peak 3 (β-carotene); Figure S2: TIC of EVOOT polyphenols after UHPLC-Q-Orbitrap analysis in negative ESI mode; Table S1: UHPLC operative setting; Table S2: Analytical parameters of phenolics identification.

## References

[1] Nowak Z.B. (2014). Folklore, fakelore, history: Invented tradition and the origins of the pizza margherita. Food Cult. Soc..

[2] European Commission (2010). Regulation No 97/2010 of February 4 2010 entering name in the register of traditional specialties guaranteed [Pizza Napoletana(S.T.G.)]. Off. J. Eur. Union.

[3] (2016). MiPAAF Ministero Politiche Agricole Alimentari e Forestali 16th Revision of List “Prodotti Agroalimentari Tradizionali”, Gazzetta Ufficiale n.143 del 21 Giugno 2016. https://www.politicheagricole.it/flex/cm/pages/ServeBLOB.php/L/IT/IDPagina/10241.

[4] Trichopoulou A., Vasilopoulou E., Georga K., Soukara S., Dilis V. (2006). Traditional foods: Why and how to sustain them. Trends Food Sci. Technol..

[5] Trichopoulou A., Soukara S., Vasilopoulou E. (2007). Traditional foods: A science and society perspective. Trends Food Sci. Technol..

[6] Fabiani R., Rosignoli P., De Bartolomeo A., Fuccelli R., Servili M., Montedoro G.F., Morozzi G., Oxidative D.N.A. (2008). Damage is prevented by extracts of olive oil, hydroxytyrosol, and other olive phenolic compounds in human blood mononuclear cells and HL60 cells. J. Nutr..

[7] Tundis R., Loizzo M., Menichini F., Statti G., Menichini F. (2008). Biological and pharmacological activities of iridoids: Recent developments. Mini Rev. Med. Chem..

[8] Dini I., Laneri S. (2019). Nutricosmetics: A brief overview. Phytother. Res..

[9] Goodwin T.W. (1954). The carotenoids of the flower petals of Calendula officinalis. Biochem. J..

[10] Drewnowski A. (2009). Sensory properties of fats and fat replacements. Nutr. Rev..

[11] Sandrou D.K., Arvanitoyannis I.S. (2000). Low-fat/calorie foods: Current stateand perspectives. Crit. Rev. Food Sci. Nutr..

[12] Crespo M.C., Tomé-Carneiro J., Dávalos A., Visioli F. (2018). Pharma-Nutritional Properties of Olive Oil Phenols. Transfer of New Findings to Human Nutrition. Foods.

[13] Zarrouk A., Martine L., Grégoire S., Nury T., Meddeb W., Camus E., Badreddine A., Durand P., Namsi A., Yammine A. (2019). Profile of fatty acids, tocopherols, phytosterols and polyphenols in Mediterranean oils (argan oils, olive oils, milk thistle seed oils and nigella seed oil) and evaluation of their antioxidant and cytoprotective activities. Curr. Pharm. Des..

[14] Obied H.K. (2013). Biography of biophenols: Past, present and future. Funct. Foods Health Dis..

[15] De Alzaa F., Guillaume C., Ravetti L. (2018). Evaluation of chemical and physical changes in different commercial oils during heating. Acta Sci. Nutr. Health.

[16] Zhang Q., Saleh S.M., Cheng J., Shen Q. (2012). Chemical alterations taken place during deep-fat frying based on certain reaction products: A review. Chem. Phys. Lipids.

[17] Dini I., Graziani G., Fedele F.L., Sicari A., Vinale F., Castaldo L., Ritieni A. (2020). Effects of Trichoderma biostimulation on the phenolic profile of extra-virgin olive oil and olive oil by-products. Antioxidants.

[18] Dini I., Seccia S., Senatore A., Coppola D., Morelli E. (2020). Development and Validation of an Analytical Method for Total Polyphenols Quantification in Extra Virgin Olive Oils. Food Anal. Methods.

[19] Soriguer F., Rojo-Martínez G., Dobarganes M.C., García Almeida J.M., Esteva I., Beltrán M., Ruiz De Adana M.S., Tinahones F., Gómez-Zumaquero J.M., García-Fuentes E. (2003). Hypertension is related to the degradation of dietary frying oils. Am. J. Clin. Nutr..

[20] Rodrigo R., Prat H., Passalacqua W., Araya J., Guichard C., Bachler J.P. (2007). Relationship be-tween oxidative stress and essential hypertension. Hypertens. Res..

[21] Ono Y., Mizuno K., Takahashi M., Miura Y., Watanabe T. (2013). Suppression of advanced glycation and lipoxidation end products by Angiotensin II type-1 receptor blocker candesartan in type 2 diabetic patients with essential hypertension. Fukushima J. Med. Sci..

[22] Baradaran A., Nasri H., Rafieian-Kopaei M. (2014). Oxidative stress and hypertension: Possibility of hypertension therapy with antioxidants. J. Res. Med. Sci..

[23] Adam S.K., Das S., Soelaiman I.N., Umar N.A., Jaarin K. (2008). Consumption of repeatedly heated soy oil increases serum parameters related to atherosclerosis in ovariectomized rats. Tohoku J. Exp. Med..

[24] Adam S.K., Das S., Jaarin K.A. (2009). detailed microscopic study of the changes in the aorta of experimental model of postmenopausal rats and with repeatedly heated palm oil. Int. J. Exp. Pathol..

[25] Ng C.Y., Leong X.F., Masbah N., Adam S.K., Yusof K., Jaarin K. (2014). Heated vegetable oils and cardiovascular risk factors. Vasc. Pharmacol..

[26] Mathias K.S., Russel G.F. (2002). Effects of processing on tomato bioactive volatile compounds. Bioact. Compd. Foods.

[27] Gahler S., Konrad O., Bohm V. (2003). Alterations of vitamin C, total phenolics, and antioxidant capacity as affected by processing tomatoes to different products. J. Agric. Food Chem..

[28] Manzo N., Santini A., Pizzolongo F., Aiello A., Romano R. (2018). Degradation kinetic (D100) of lycopene during the thermal treatment of concentrated tomato paste. Nat. Prod. Res..

[29] Grabowska M., Wawrzyniak D., Rolle K., Chomczynski P., Oziewicz S., Jurgaand S., Barciszewski J. (2019). Let food be your medicine: Nutraceutical properties of lycopene. Food Funct..

[30] Associazione Verace Pizza Napoletana. https://www.pizzanapoletana.org/images/file/disciplinare%202008%20UK.pdf.

[31] Gao X., Bjork L., Trajkovski V., Uggla M. (2000). Evaluation of antioxidant activities of rosehip ethanol extracts in different test systems. J. Agric. Food Chem..

[32] Re R., Pellegrini N., Proteggente A., Pannala A., Yang M., Rice Evans C. (1999). Antioxidant activity applying an improved ABTS radical cation decolorization assay. Free Rad. Biol. Med..

[33] Minekus M., Alminger M., Alvito P., Ballance S., Bohn T., Bourlieu C., Carriere F., Boutrou R., Corredig M., Dupont D. (2014). A standardised static in vitro digestion method suitable for food–an international consensus. Food Funct..

[34] Raiola A., Meca G., Mañes J., Ritieni A. (2012). Bioaccessibility of Deoxynivalenol and its natural co-occurrence with Ochratoxin A and Aflatoxin B1 in Italian commercial pasta. Food Chem. Toxicol..

[35] Weichselbaum E., Benelam B., Soares Costa H. (2009). Traditional Foods in Europe.

[36] Durazzo A., Lisciani S., Camilli E., Gabrielli P., Marconi S., Gambelli L., Aguzzi A., Lucarini M., Maiani G., Casale G. (2017). Nutritional composition and antioxidant properties of traditional Italian dishes. Food Chem..

[37] Dini I., Graziani G., Gaspari A., Fedele F.L., Sicari A., Vinale F., Cavallo P., Lorito M., Ritieni A. (2020). New Strategies in the Cultivation of Olive Trees and Repercussions on the Nutritional Value of the Extra Virgin Olive Oil. Molecules.

[38] Scalbert A., Williamson G. (2000). Dietary Intake and Bioavailability of Polyphenols. Nutr. J..

[39] Cavallo P., Dini I., Sepe I., Galasso G., Fedele F.L., Sicari A., Bolletti Censi S., Gaspari A., Ritieni A., Lorito M. (2020). An Innovative Olive Pâté with Nutraceutical Properties. Antioxidants.

[40] Caporaso N., Panariello V., Sacchi R. (2015). The “true” Neapolitan pizza: Assessing the influence of extra virgin olive oil on pizza volatile compounds and lipid oxidation. J. Culin. Sci. Technol..

[41] Miro-Casas E., Covas M., Farre M., Fito M., Ortuño J., Weinbrenner T., Roset P., De la Torre R. (2003). Hydroxytyrosol disposition in humans. Clin. Chem..

[42] Bardhan K.D., Strugala V., Dettmar P.W. (2012). Reflux Revisited: Advancing the Role of Pepsin. Int. J. Otolaryngol..

[43] Rubió L., Serra A., Oliver Chen C.-Y., Macià A., Covas M.I., Sola R., Motilva M.J. (2014). Effect of the cooccurring components from olive oil and thyme extracts on the antioxidant status and its bioavailability in an acute ingestion in rats. Food Funct..

[44] Müller L., Caris-veyrat C., Lowe G., Böhm V. (2016). Lycopene and its antioxidant role in the prevention of cardiovascular diseases—A critical review. Crit. Rev. Food Sci. Nutr..

[45] El-raey M.A., Ibrahim G.E., Eldahshan O.A. (2013). lycopene and lutein; a review for their chemistry and medicinal uses. J. Pharmacogn. Phytochem..

[46] Holzenburg J.A., King S. (2012). Physical barriers to carotenoid bioaccessibility Ultrastructure survey of chromoplast and cell wall morphology in nine carotenoid-containing fruits and vegetables. J. Sci. Food Agric..

[47] Rich G.T., Bailey A.L., Faulks R.M., Parker M.L., Wickham M.S.J., Fillery-Travis A. (2003). Solubilization of carotenoids from carrot juice and spinach in lipid phases: I. Modeling the gastric lumen. Lipids.

[48] Thane C., Reddy S. (1997). Processing of fruit and vegetables: Effect on carotenoids. Food Sci. Nutr..

[49] Dewanto V., Wu X., Adom K.K., Liu R.H. (2002). Thermal processing enhances the nutritional value of tomatoes by increasing total antioxidant activity. J. Agric. Food Chem..

[50] Nicoli M.C., Anese M., Parpinel M. (1999). Influence of processing on the antioxidant properties of fruit and vegetables. Trends Food Sci. Technol..

[51] Pinelo M., Manzocco L., Nuñez M.J., Nicoli M.C. (2004). Interaction among phenols in food fortification: Negative synergism on antioxidant capacity. J. Agric. Food Chem..

[52] Yu J., Gleize B., Zhang L., Caris-Veyrat C., Renard C.M.G.C. (2019). Heating tomato puree in the presence of lipids and onion: The impact of onion on lycopene isomerization. Food Chem..

[53] De Alvarenga J.F.R., Tran C., Hurtado-Barroso S., Martinez-Huélamo M., Illan M., Lamuela-Raventos R.M. (2017). Home cooking and ingredient synergism improve lycopene isomer production in Sofrito. Food Res. Int..

[54] Orsavova J., Misurcova L., Ambrozova J., Vicha R., Mlcek J. (2015). Fatty acids composition of vegetable oils and its contribution to dietary energy intake and dependence of cardiovascular mortality on dietary intake of fatty acids. Int. J. Mol. Sci..

[55] Medina E., De Castro A., Romero C., Brenes M. (2006). Comparison of the Concentrations of Phenolic Compounds in Olive Oils and Other Plant Oils: Correlation with Antimicrobial Activity. J. Agric. Food Chem..

[56] Sandberg A.S. (2005). Methods and options in vitro dialyzability; benefits and limitations. Int. J. Vitam. Nutr. Res..

[57] Colle I.J.P., Lemmens L., Van Buggenhout S., Met K., Van Loey A.M., Hendrickx M.E. (2013). Processing tomato pulp in the presence of lipids: The impact on lycopene bioaccessibility. Food Res. Inter..

[58] Porter C.J.H., Kaukonen A.M., Taillardat-Bertschinger A., Boyd B.J., O’Connor J.M., Edwards G.A., Edwards G.A., Charman W.N. (2004). Use of in vitro lipid digestion data to explain the in vivo performance of triglyceride-based oral lipid formulations of poorly water-soluble drugs: Studies with halofantrine. J. Pharm. Sci..

[59] Yu J., Gleize B., Zhang L., Caris-Veyrat C., Renard C.M.G.C. (2019). A D-optimal mixture design of tomato-based sauce formulations: Efects of onion and EVOO on lycopene isomerization and bioaccessibility. Food Funct..

[60] Honda M., Horiuchi I., Hiramatsu H., Inoue Y., Kitamura C., Fukaya T., Takehara M. (2016). Vegetable oilmediated thermal isomerization of (all-*E*)-lycopene: Facile and efficient production of *Z*-isomers. Eur. J. Lipid Sci. Technol..

[61] Colle I.J.P., Lemmens L., Tolesa G.N., Van Buggenhout S., De Vleeschouwer K., Van Loey A.M., Hendrickx M.E. (2010). Lycopene Degradation and Isomerization Kinetics during Thermal Processing of an Olive Oil/Tomato Emulsion. J. Agric. Food Chem..

[62] Colle I.J.P., Van Buggenhout S., Lemmens L., Van Loey A.M., Hendrickx M.E. (2012). The type and quantity of lipids present during digestion influence the in vitro bioaccessibility of lycopene from raw tomato pulp. Food Res. Int..

[63] Tulipani S., Martinez Huelamo M., Rotches Ribalta M., Estruch R., Ferrer E.E., Andres-Lacueva C., Illan M., Lamuela-Raventós R.M.L. (2012). Oil matrix effects on plasma exposure and urinary excretion of phenolic compounds from tomato sauces: Evidence from a human pilot study. Food Chem..

