# Supplementary material for: The Nutraceutical Properties of “Pizza Napoletana Marinara TSG” a Traditional Food Rich in Bioaccessible Antioxidants
Source: Antioxidants (Basel). 2021 Mar 22;10(3):495. doi: 10.3390/antiox10030495 (PMC8004925; doi:10.3390/antiox10030495)
Supplement: Supplementary file 1 [file antioxidants-10-00495-s001.pdf]

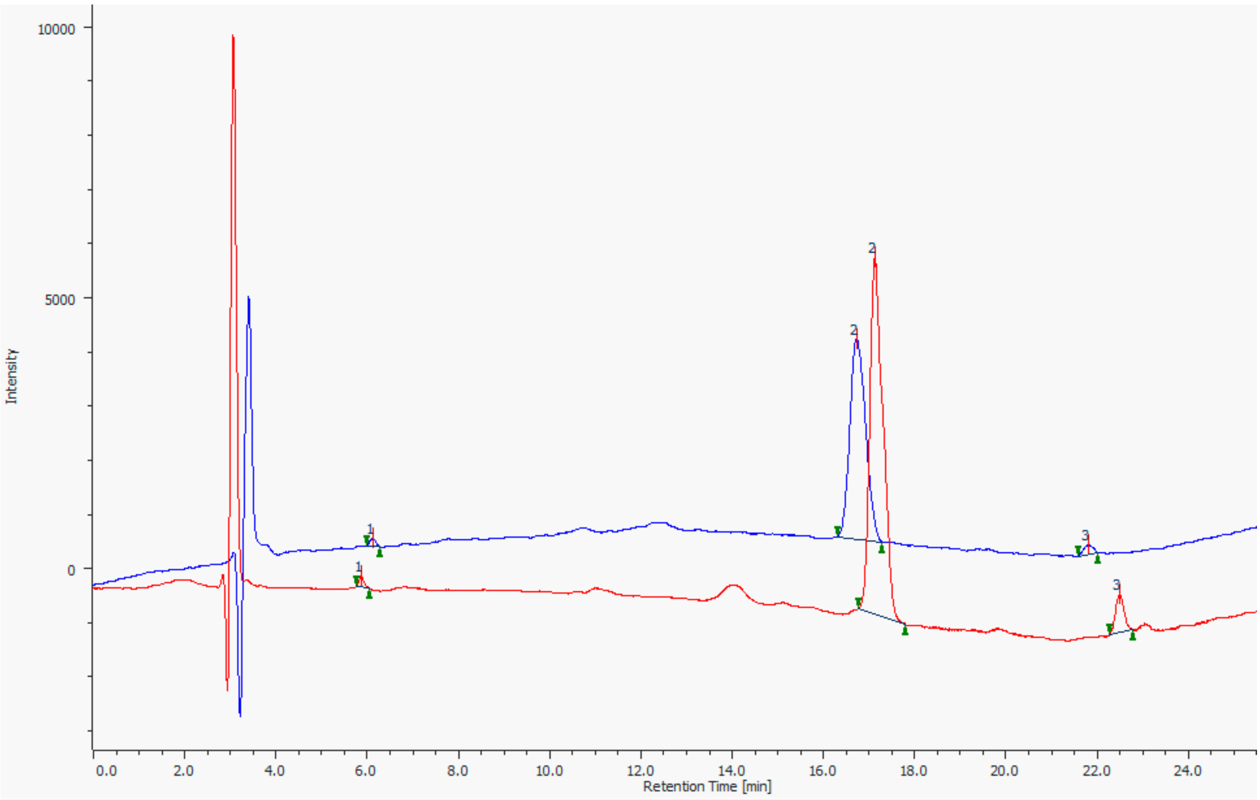

Figure S1. Comparison from carotenoids profile of tomato sauce and cherry tomato: peak 1 (lutein), peak 2 (lycopene), peak 3 (β-carotene).

Table S1. UHPLC operative setting

**UHPLC** Ultra-High-Performance Liquid Chromatography, Thermo Fisher Scientific, Waltham				**Mass operative setting** Orbitrap LC-MS/MS (Q Exactive, Waltham, MA, USA)			
**Stationary phase**	**Mobile phase**			**Ion source parameters** HESI II (Thermo Fisher Scientific, Waltham, MA, USA)		**Analyzer Target SIM**	
Accucore aQ 2.6 µm 100 × 2.1 mm column (Thermo Scientific, Waltham, USA) in a thermostat column compartment (T = 30 °C)	**Minutes**	**Phase A% acetic acid (0.1%)**	**Phase B% 100% ACN**				
	0-5	95	5	Spray voltage	-3.0 kV	automatic gain control (AGC)	1 × 10^5 ions
	6-25	60	40	Sheath gas	(N_2 > 95%)	Scan rate	$2s^{-1}$
	25.1-27	0	100	Auxiliary gas	(N_2 > 95%)	Scan range	100-1500 m/z
	27.1-35	95	5	Capillary temperature	200 °C	mass resolving power	35,000 full width at half maximum (at *m/z* 200);
						Maximum injection	200 ms
	35.1-45	100	0	S-lens	RF level 50		
				Auxiliary gas heater temperature	305°C.	The SIM (selected ion monitoring acquisition) parameters were: 35,000 full widths and half maximum (at *m/z* 200) (resolution power); 15s (time window); 1.2 *m/z* (quadrupole isolation window	

Table S2. Analytical parameters of phenolics identification

	Phenolics	RT (min)	Formula	Theoretical m/z of deprotonated molecular ions [M-H]$^-$	Experimental m/z [M-H]$^-$	Calculated errors Δppm	Fragments	Collision energy (eV)
Phenolic acids	Vanillic acid	4.26	$C_8H_8O_4$	167.03498	167.03522	1.44	152.01143	20
	p-Coumaric acid	9.69	$C_9H_{10}O_5$	163.04007	163.04028	1.29	119.05023	20
	Cinnamic acid	11.39	$C_9H_8O_2$	147.04515	147.04536	1.43	103.04501	20
	Ferulic acid	12.36	$C_{10}H_{10}O_4$	193.05063	193.05084	1.09	178.02685	20
	4-Hydroxybenzoic acid	2.58	$C_7H_6O_3$	137.02442	137.02456	1.02	93.03431	12
	3-Hydroxybenzoic acid	2.88	$C_7H_6O_3$	137.02442	137.02458	1.17	93.03431	12
Flavonoids	Apigenin	19.11	$C_{15}H_{10}O_5$	269.04555	269.04597	1.56	225.05592	35
	Luteolin	19.07	$C_{15}H_{10}O_6$	285.04046	285.04106	2.10	133.02940	30
Lignans	(+) Pinoresinol	17.00	$C_{20}H_{22}O_6$	357.13436	357.13487	1.43	151.03961	40
	(+) 1-Acetoxypinoresinol	18.86	$C_{22}H_{24}O_8$	415.13984	415.14007	0.55	415.13821	40
Phenolic alcohols	Hydroxytyrosol (3,4 DHPEA)	1.58	$C_8H_{10}O_3$	153.05572	153.05580	0.52	123.04561	12
	Tyrosol (*p*-HPEA)	2.57	$C_8H_{10}O_2$	137.06080	137.06096	1.17	119.05022	12
Secoiridoids	Elenaic acid	13.11	$C_{11}H_{14}O_6$	241.07176	241.07212	1.49	209.04573	10
	Oleacein (3.4 DHPEA-EDA)	16.12	$C_{17}H_{20}O_6$	319.11871	319.11898	0.85	301.1082	15
	Oleuropein	16.74	$C_{25}H_{32}O_{13}$	539.17701	539.17767	1.22	377.12393	20
	Ligstroside	18.37	$C_{25}H_{32}O_{12}$	523.18210	523.18279	1.32	361.12914	12
	Ligstroside-aglycone dialdehyde (*p*-HPEA-EDA)	18.61	$C_{17}H_{20}O_5$	303.12380	303.12441	2.01	301.1082	12
	Secologanoside	19.49	$C_{16}H_{21}O_{11}$	389.1092	389.109258	0.59	345.1195	12
	Oleuropein-aglycone mono-aldehyde (3.4 DHPEA-EA)	21.24	$C_{19}H_{22}O_8$	377.12419	377.12442	0.61	345.09790	12

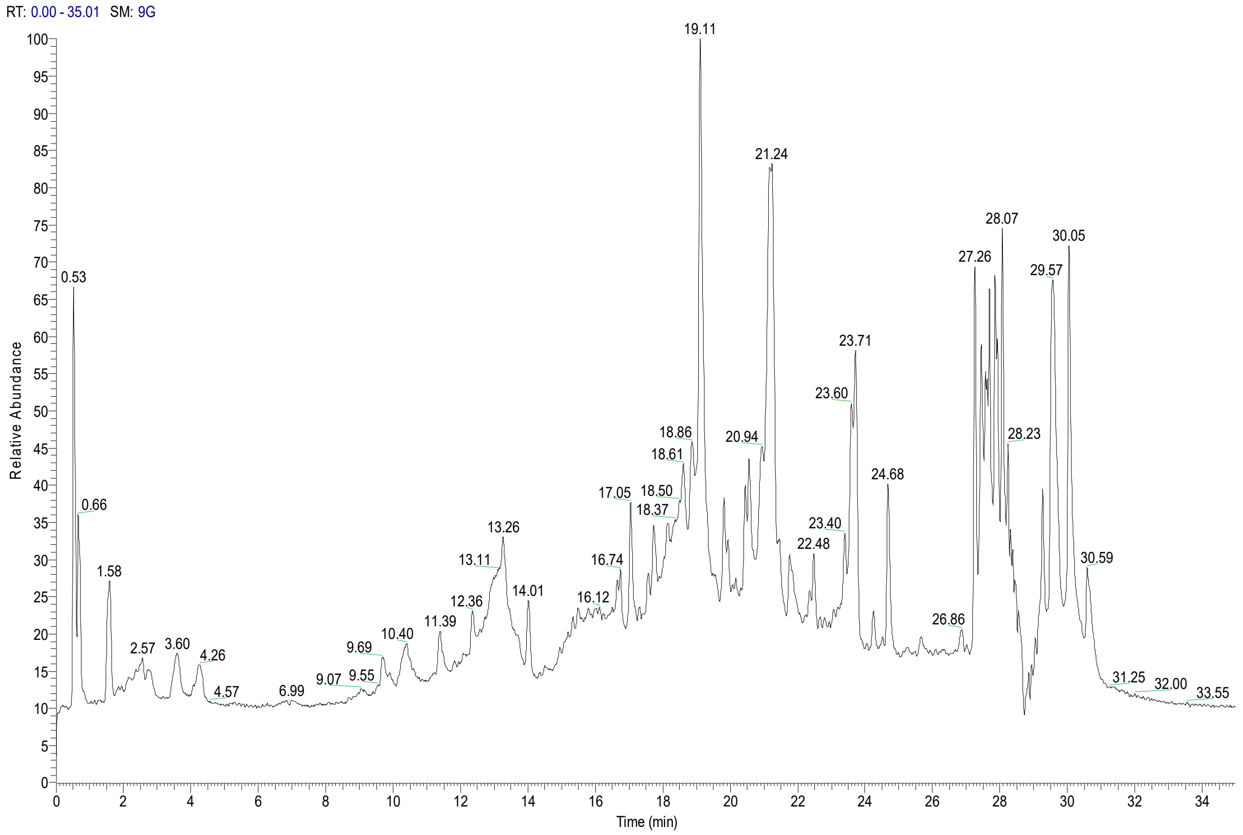

Figure S2: TIC of EVOOT polyphenols after UHPLC-Q-Orbitrap analysis in negative ESI mode
